# Simultaneous detection of bovine and porcine DNA in pharmaceutical gelatin capsules by duplex PCR assay for Halal authentication

**DOI:** 10.1186/s40199-017-0171-3

**Published:** 2017-02-14

**Authors:** Jafar Nikzad, Soraya Shahhosseini, Maryam Tabarzad, Nastaran Nafissi-Varcheh, Maryam Torshabi

**Affiliations:** 1 School of Pharmacy, Shahid Beheshti University of Medical Sciences, Tehran, Iran; 2 Department of Pharmaceutical Chemistry, School of Pharmacy, Shahid Beheshti University of Medical Sciences, Tehran, Iran; 3 Protein Technology Research Center, Shahid Beheshti University of Medical Sciences, Tehran, Iran; 4 Department of Pharmaceutical Biotechnology, School of Pharmacy, Shahid Beheshti University of Medical Sciences, Tehran, Iran; 5 Department of Dental Biomaterials, School of Dentistry, Shahid Beheshti University of Medical Sciences, Tehran, Iran

**Keywords:** Gelatin, Capsule, Bovine, Porcine, Duplex PCR

## Abstract

**Background:**

In the pharmaceutical industry, hard- and soft-shelled capsules are typically made from gelatin, commonly derived from bovine and porcine sources. To ensure that pharmaceutical products comply with halal regulations in Muslim countries (no porcine products allowed), development of a valid, reliable, quick, and most importantly, cost-effective tests are of utmost importance.

**Methods:**

We developed a species-specific duplex polymerase chain reaction (PCR) assay targeting 149 bp porcine and 271 bp bovine mitochondrial DNA (mtDNA) to simultaneously detect both porcine and bovine DNA (in one reaction at the same time) in gelatin. Some additional simplex PCR tests (targeting 126 bp bovine and 212 bp porcine mtDNA) and real-time PCR using a commercially available kit (for identification of porcine DNA) were used to verify the selectivity and sensitivity of our duplex PCR. After optimization of DNA extraction and PCR methods, hard/soft pharmaceutical gelatin capsules (containing drug) were tested for the presence of porcine and/or bovine DNA.

**Results:**

Duplex PCR detected the presence of as little as 0.1% porcine DNA, which was more accurate than the commercially available kit. Of all gelatin capsules tested (*n* = 24), 50% contained porcine DNA (pure porcine gelatin alone or in combination with bovine gelatin).

**Conclusions:**

Duplex PCR presents an easy-to-follow, quick, low-cost and reliable method to simultaneously detect porcine and bovine DNAs (>100 bp) in minute amounts in highly processed gelatin-containing pharmaceutical products (with a 0.1% sensitivity for porcine DNA) which may be used for halal authentication.

**Graphical abstract:**

Simultaneous detection of porcine and bovine DNA in gelatin capsules by duplex PCR 
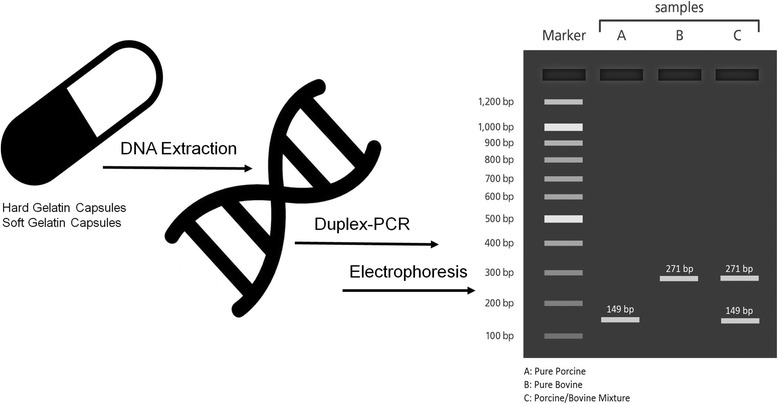

## Background

Halal foods (in Islam), pertains to the lawful (or blessed) food or non-food products including pharmaceuticals. While food products are strictly monitored during halal certification, there are no such requirements for non-food products (i.e., pharmaceuticals). Gelatin is a high molecular weight protein that is widely used as a viscous agent in hard and soft capsules. Soft capsules are mainly filled with liquids, while hard capsules are filled with powder, and vary both in composition and production processes [1]. Gelatin is produced from partial denaturation of collagen extracted from the skin, bone, and connective tissue of animals (i.e., cattle and pigs) [2]. Most (90%) gelatin capsules are derived from porcine tissues due to greater strength, resistance to stress, ability to hold water, higher melting point, shorter production time (30 days versus 60–80 days for bovine gelatin), and low cost [3, 4].

Identifying the source of gelatin is of importance due both to concerns regarding possible disease transmission to humans, as well as religious concerns in Muslim countries (which strictly forbid porcine products) [5–7]. Methods that rely on physicochemical properties (i.e., chemical precipitation and Fourier transform infrared spectroscopy) have been proven unsuitable for differentiating a mixture of gelatin (i.e., bovine/porcine mixtures) mainly because of the similarities in structure and physicochemical properties of gelatin derived from different sources [8]. There are a number of molecular techniques that can be used to identify the origin of gelatin products such as protein/antibody-based (i.e., high-performance liquid chromatography and enzyme-linked immunosorbent assays) [7, 9–13] and DNA-based techniques. It is reported that protein-based analytical techniques for the species identification in mixed samples are significantly less sensitive than DNA-based techniques for evaluation of thermally processed materials (i.e., gelatin) because of specific epitope alterations [8, 14, 15]. The methods used for the processing and production of gelatin include acid/base connective tissue hydrolysis, high-temperature extraction using water and sterilization. Hence, gelatin contains very small amounts of highly degraded DNA [16]. In fact, DNA is a relatively stable molecule, which can better withstand heat processing and can be detect even though it will be in fragmented form [14]. DNA detection can help scientists and regulation agencies detect impurities and identify the origin of gelatin products [17]. This varies from material to material. A heightened sensitivity is therefore required in order to detect impurities within products.

Detection and quantification of trace DNA can be performed using polymerase chain reaction (PCR)-based methods which have had the greatest success due to higher sensitivity, specificity, rapidity, and reproducibility. On the other hand, extraction of high-quality DNA is an important prerequisite for PCR-based techniques, which could be a potential problem if there is extensive damage to DNA following heat processing [18, 19]. Many primers have been developed based on both mitochondrial and nuclear genes to trace species-specific DNA. Mitochondrial DNA analysis using PCR offers a series of advantages. The mtDNA genes are present in thousands of copies per cell; thus, the large variability of mtDNA allows reliable identification of precise species in mixtures. Although nuclear DNA (linear) is more powerful, mtDNA (circular) is more stable over time/and may also present intracellularly. The mtDNA of most animals codes for 37 genes; one of which is the gene for cytochrome b (Cyt b) [19, 20]. There are numerous articles related to detection of porcine or bovine DNA in foods; but up to now only few have used PCR methodology [i.e., conventional PCR, real-time PCR, PCR-southern hybridization and PCR-restriction fragment length polymorphism (RFLP)] to detect porcine or bovine DNA in gelatin capsule shells [4, 6, 9, 21–23].

Multiplex PCR (i.e., duplex, triplex, etc.) is a widespread molecular biology technique for simultaneous amplification/detection of multiple targets (with a different pair of primers for each target in the same reaction tube) in a single PCR experiment. Multiplex PCR has a number of advantages. It provides more information by using fewer initial samples, it is cost effective and saves times (fewer reagents/steps) and it is highly accurate (fewer errors, improved data normalization). Since bovine (the most widely used type of gelatin in Muslim countries) and porcine gelatin are the most commonly used types of gelatin in the production of pharmaceutical capsules, simultaneous detection of both bovine and porcine can be useful, time-saving, and cost-effective. The purpose of this study was to introduce a suitable and sensitive technique to simultaneously detect bovine and porcine DNA in gelatin-containing products especially in soft and hard gelatin drug-containing capsules.

## Methods

### Sample preparation

Pure bovine (180–200 g) and porcine (G2500) gelatin powders were purchased (from Faravari Darooie Gelatin Halal, Iran and Sigma-Aldrich, Germany, respectively) and used. Gelatin standard mixtures were prepared by adding 0.1, 1, 10, 50 and 75% w/w porcine gelatin powder to bovine gelatin powder (Table 2). A total of 24 pharmaceutical hard (*n* = 12) and soft (*n* = 12) gelatin capsules (containing drug) from different national (*n* = 8) and international (*n* = 16) companies were purchased (2015–2016) from pharmacies in Tehran (Iran) and assessed (Table 3).

### DNA extraction

DNA was extracted from pure gelatin powder (100% w/w bovine or porcine), binary mixtures of bovine/porcine gelatin powder (0.1–75% w/w) and pharmaceutical capsules using a column-based DNeasy Mericon Food Kit (Qiagen, Germany). The extraction process (small fragment protocol on 200 mg of initial sample) was performed under DNA contamination/degradation-free conditions in order to minimize pseudo-results (from reagent and laboratory environment contamination and especially from cross-contamination between samples) and inhibit the degradation of extracted DNA (by environmental DNases), respectively. First, the drug contents of the capsules (powder or liquid) were emptied and soft capsule shells were washed with autoclaved ultra violet-treated (UV) deionized water. Subsequently, the shells were minced using 10% bleach and UV-treated scissors; then 200 mg were transferred into a 2 mL sterile DNase-free microcentrifuge tube. The DNA extraction was performed according to the manufacturer’s instructions with some modifications to maximize recovery of short DNA fragments. First, 1 mL of lysis buffer and 25 μL of proteinase K solution were added to the tubes (containing 200 mg of the standard powder or minced capsule shells) and were incubated for 30 min at 60 °C in a dry bath incubator (INC-13, NAMSA, USA). Subsequently, the solution was cooled to room temperature on ice for approximately 15 min and centrifuged (Hettich, Germany) for 5 min at 2,500 g. Subsequently, the clear supernatant (700 μL) was transferred to a new 2 mL microcentrifuge tube, which contained 500 μL of chloroform (Merck, Germany), and was centrifuged at 14,000 g for 15 min. Upon completion, 250 μL of the upper aqueous phase was added to a fresh 2 mL microcentrifuge tube containing 1 mL of binding buffer and was thoroughly hand-mixed. To achieve a higher DNA yield, this step was repeated with another 250 μL of the upper aqueous phase (500 μL of the upper aqueous fluid). Next, 600 μL of the mixture was pipetted into a spin column and was placed in a 2 mL collection tube and centrifuged at 17,900 g for 2 min; then the flow-through was subsequently discarded. This step was repeated 2 more times (600 μL in total) in order to increase the yield of the extracted DNA. Afterwards, 500 μL of the wash buffer was added to the spin column and centrifuged at 17,900 g for 2 min; then the flow-through was discarded. The collection tube was centrifuged again at 17,900 g for 4 min to dry the membrane. Finally, the spin column was transferred to a fresh 1.5 mL microcentrifuge tube, and 30 μL of elution buffer was added onto the membrane and incubated for 5 min at room temperature; and then centrifuged again at 17,900 g for another 2 min. This process was repeated once more. The DNA extraction process was repeated twice for each gelatin sample. We ran the extraction process on the lysis buffer alone (which did not contain any gelatin), as the negative control (blank). The extracted DNA solutions were stored at -20 °C for further analysis.

### DNA quantification and purity

The quality and quantity of the extracted DNA material were determined by spectrophotometry, using a NanoDrop™ 2000/2000c spectrophotometer (Thermo Scientific, USA). DNA concentration was determined by UV absorbance at 260 nm and purity of the extracted DNA was determined by the ratio of absorbance at 260 and 280 nm.

### Simplex and duplex PCR

Four sets of specific primers for bovine and porcine mtDNA that were used for PCR amplification (Bioneer, South Korea) are listed in Table 1 [24–27]. Simplex PCR amplifications were carried out in 20 μL of total reaction volume containing 2 μL of DNA extract, 10 μL of Taq DNA polymerase master mix red (1.5 mM Mg^2+^) (Ampliqon, Denmark), and only one pair of forward and reverse primers (0.4 μM of 149-F/-R porcine primer, 0.15 μM of 271-F/-R bovine primer, 0.2 μM of 126-F/-R bovine primer or 0.2 μM of 212-F/-R porcine primer), and nuclease-free water to adjust the volume (CinnaGen, Iran). Duplex PCR amplifications were carried out as described above with some differences. Total duplex PCR reaction volume (20 μL) contained two pairs of primers with different final concentrations of 0.35 μM of 149-F/-R porcine primer and 0.125 μM of 271-F/-R bovine primer. Reactions without DNA template (NTC) and with 2 μL of DNA extraction negative controls (blanks) were used for each primer pair and checked for DNA contamination in PCR amplifications and DNA extraction processes respectively. Polymerase chain reaction amplifications were performed in a thermal cycler (PeQlab, Germany) under the following conditions: Initial denaturation at 94 °C for 2 min followed by 35 cycles of denaturation at 94 °C for 30 s, 60 °C for 30 s, and 72 °C for 30 s. Final extension was carried out at 72 °C for 5 min. Each reaction was repeated at least twice for each DNA sample at different times.

**Table 1:** Species-specific oligonucleotide primers used in this study

Species	Primer Sequences (5′ - > 3′)	Target Gene	Annealing Temperature (°C)	Amplicon (bp)	Reference
Porcine	F: ATGAAACATTGGAGTAGTCCTACTATTTACC	Cyt b	60	149	[24]
	R: CTACGAGGTCTGTTCCGATATAAGG				
	F: GCCTAAATCTCCCCTCAATGGTA	Cyt b	60	212	[25]
	R: ATGAAAGAGGCAAATAGATTTTCG				
Bovine	F: ATGATCTTATCAATATTCTTGACCC	ATPase 8	60	126	[26]
	R: CCTTCAAGGGGTGTTTTGTTTTAA				
	F: GCCATATACTCTCCTTGGTGACA	Cyt b	60	271	[27]
	R: GTAGGCTTGGGAATAGTACGA				

### Gel electrophoresis and semi-quantitative analysis

Amplified PCR products were analyzed using 2% agarose gel in 0.5X Tris–acetate–ethylene diamine tetra acetic acid (TAE) buffer and DNA safe stain (Parstus, Iran) as a visualizing agent (ran for 45 min at 100–120 V). All agarose gels in the above experiment used 100 bp DNA ladder (Parstus, Iran) as the size marker and were visualized using UV transilluminator gel documentation (Vilber Lourmat, France); digital images were obtained. The conventional duplex PCR (qualitative) assay was then optimized for a semi-quantitative approach to analyze PCR band intensities after agarose gel electrophoresis using the Scion Image software (ScnImage.exe) (Scion corporation, Maryland). Briefly, fluorescence intensities of the obtained PCR bands from simultaneous amplification of bovine and porcine DNAs from different mixtures of bovine and porcine standard powders were normalized. The normalized band intensities for porcine PCR products were calculated using the following expression: N_porcine_ = I_porcine_/(I_porcine_ + I_bovine_), where N_porcine_ is the normalized band intensity for porcine DNA, and I_porcine_ and I_bovine_ are the band intensities for porcine and bovine DNAs, respectively [15].

### Real-time PCR using the mericon pig kit

In addition to simplex PCR (porcine-212 and bovine-126 bp amplicons), we used the commercial Mericon Pig Kit (Qiagen, Germany) to confirm the developed duplex PCR (porcine-149/bovine-271 bp amplicons) in this study. The assay uses a real-time PCR-based protocol. The amplifications of this real-time PCR-based protocol were carried out in 20 μL of total reaction volume containing 9.6 μL of sample DNA (2 μL of DNA extract from the standard powders or pharmaceutical capsules and 7.6 μL of QuantiTect nucleic acid dilution buffer) and 10.4 μL of reconstituted Mericon assay, containing multiplex PCR master mix HotStarTaq®Plus DNA polymerase, multiplex real-time PCR buffer and dNTP), Mericon assay contained target specific primers and probes and internal control and ROX dye. In some samples with faint porcine bands in duplex PCR but no positive results using the kit, the reactions were repeated with 4 and 9.6 μL of extracted DNA. Amplifications were performed on the ABI StepOne™ detection system (Applied Biosystem Instruments, USA) using the following thermal cycling conditions: pre-PCR stage at 60 °C for 30 s, denaturation at 95 °C for 10 min (holding stage), followed by 45 cycles of denaturation at 95 °C for 15 s, annealing and extension at 60 °C for 1 min (cycling stage), and post-PCR stage at 60 °C for 30 s. According to the kit instructions, FAM (fluorescein) and VIC channels were used to detect target DNA (porcine) and the internal control (in order to confirm successful PCR), respectively. Each experiment was repeated at least 3 times for each DNA template. Reactions without DNA template (NTC) and with 2 μL of DNA extraction negative controls (blanks) were used for each primer pair in order to check for DNA contamination in PCR amplifications and DNA extraction processes, respectively. Reactions with porcine DNA [9.6 μL of the positive control provided by the kit and 2 μL of DNA extract from 50% (w/w) bovine/porcine standard powder mixture] were considered as positive controls. Amplification plots of normalized fluorescent signals (delta Rn) versus cycles were analyzed using StepOne ™ software version 2.1 (Applied Biosystem Instruments, USA).

## Results

### Quantitative analysis of extracted DNA

The quantity and purity of the DNA extracts from 200 mg standard gelatin powders (Table 2) and minced hard/soft-gelatin capsule shells (from drug-containing capsules) (Table 3) were examined by spectrophotometry. The ratio of A260/A280 ranged between 1.7 and 1.8. DNA yields ranged between 6.5 and 131 ng/μL.

**Table 2:** Detection of porcine and bovine DNA in gelatin standard powder (pure bovine, pure porcine and bovine/porcine mixtures) using duplex PCR, simplex PCR (for confirmation of duplex PCR results) and real-time PCR using commercial porcine DNA detection kit (for confirmation of duplex PCR results) (2 μL extracted DNA/20 μL PCR reaction)

Gelatin (standard powder)		Extracted DNA	Duplex PCR products		Simplex PCR products		*Mericon* Pig Kit (porcine DNA)
Bovine (% w/w)	Porcine (% w/w)	Concentration (ng/μL) [260/280 (ratio)]	149 bp band (porcine)	271 bp band (bovine)	212 bp band (porcine)	126 bp band (bovine)	
100.0	0.0	131.0 [1.8]	-	+	-	+	-
99.9	0.1	118.0 [1.7]	+ (faint)	+	+ (faint)	+	-
99.0	1.0	130.5 [1.8]	+	+	+	+	- (+)^a^
90.0	10.0	109.0 [1.8]	+	+	+	+	+
50.0	50.0	86.5 [1.7]	+	+	+	+	+
25.0	75.0	52.0 [1.7]	+	+	+	+	+
0	100.0	34.0 [1.7]	+	-	+	-	+

^a^Positive with 4 μL extracted DNA/20 μL PCR reaction

**Table 3:** Detection of porcine and bovine DNA in hard (no. 1–12) and soft (no. 13–24) pharmaceutical gelatin capsules (from different companies) using duplex PCR, simplex PCR (for confirmation of duplex PCR results) and real-time PCR using commercial porcine DNA detection kit (for confirmation of duplex PCR results) (2 μL extracted DNA/20 μL PCR reaction)

Pharmaceutical gelatin capsule		Extracted DNA	Duplex PCR products		Simplex PCR products		*Mericon* Pig Kit (porcine DNA)
Number (no.)	Content	Concentration (ng/μL) [260/280 (ratio)]	149 bp band (porcine)	271 bp band (bovine)	212 bp band (porcine)	126 bp band (bovine)	
1	Omeprazole	15.5 [1.7]	-	+	-	+	-
2	Pancreatin	6.5 [1.7]	+	-	+	-	+
3	Omeprazole	18.0 [1.7]	+ (faint)	+	+	+	- (+)^a^
4	Piroxicam	53.0 [1.8]	-	+	-	+	-
5	Venlafaxin	85.0 [1.8]	-	+	-	+	-
6	Diclofenac	44.0 [1.8]	-	+	-	+	-
7	Levodopa	57.0 [1.8]	-	+	-	+	-
8	Mebeverine	93.0 [1.8]	-	+	-	+	-
9	Duloxetine	23.0 [1.8]	+ (faint)	+	+ (faint)	+	- (+)^b^
10	Clindamycin	43.0 [1.8]	-	+	-	+	-
11	Celecoxib	55.0 [1.8]	-	+	-	+	-
12	Lansoprazole	124.5 [1.8]	+	+	+	+	+
13	Ibuprofen	15.6 [1.7]	-	+	-	+	-
14	Multivitamin	10.8 [1.7]	+	-	+	-	+
15	Acetaminophen	55.0 [1.8]	+	+	+	+	+
16	Adult Cold	58.0 [1.8]	+	+	+	+	+
17	Magnesium	14.0 [1.7]	-	+	-	+	-
18	Liver Oil	15.0 [1.7]	+	-	+	-	+
19	Primrose Oil	17.0 [1.8]	-	+	-	+	-
20	Multivitamin	16.0 [1.7]	+	-	+	-	+
21	Minerals	14.0 [1.7]	+	-	+	-	+
22	Multivitamin	30.0 [1.7]	+	-	+	-	+
23	Multivitamin	22.5 [1.7]	-	+	-	+	-
24	Fish Oil	15.0 [1.7]	+	-	+	-	+
Total Positive Capsules for Porcine DNA (%)				12 (50%)			
Total Negative Capsules for Porcine DNA (%)				12 (50%)			

^a^Positive with 4 μL extracted DNA/20 μL PCR reaction, ^b^Positive with 9.6 μL extracted DNA/20 μL PCR reaction

### Simplex PCR

**Fig. 1:**
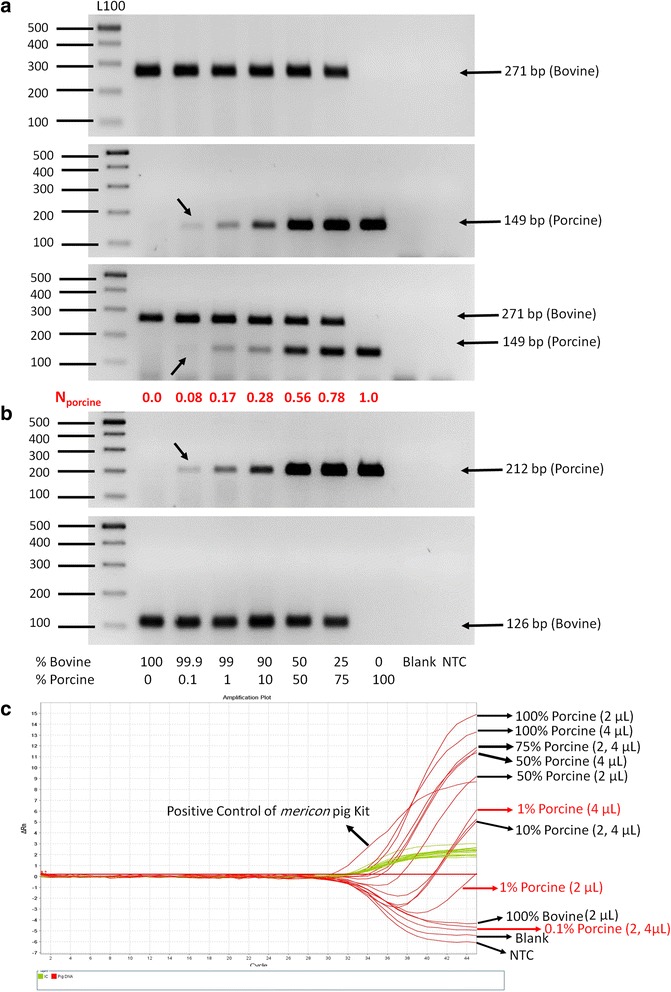
**a**: Agarose gel electrophoresis of simplex and duplex PCR products (149 bp for porcine and 271 bp for bovine mtDNA) resulting from DNA extraction (2 μL/20 μL PCR reaction) of pure bovine and porcine standard powders (100%) and reference mixtures of bovine/porcine powders [﻿0.1, 1, 10, 50 and 75% (w/w) of porcine]. L100: 100 bp ladder; Blank: Negative control of DNA extractions; NTC: Negative control of PCR reactions; N_pork_: The normalized band intensity for porcine DNA calculated with image analysis software. The faint bands are marked with arrows. **b**: Agarose gel electrophoresis of simplex PCR products (212 bp for porcine and 126 bp for bovine mtDNA) of above DNA samples (2 μL) for confirmation of duplex PCR results. **c**: Amplification plot (Delta Rn *vs* Cycle) of real-time PCR of the above DNA samples (2 and 4 μL) using *mericon* pig kit for confirming duplex PCR results. Red and green curves display target DNA (porcine) and internal control (IC), respectively

**Fig. 2:**
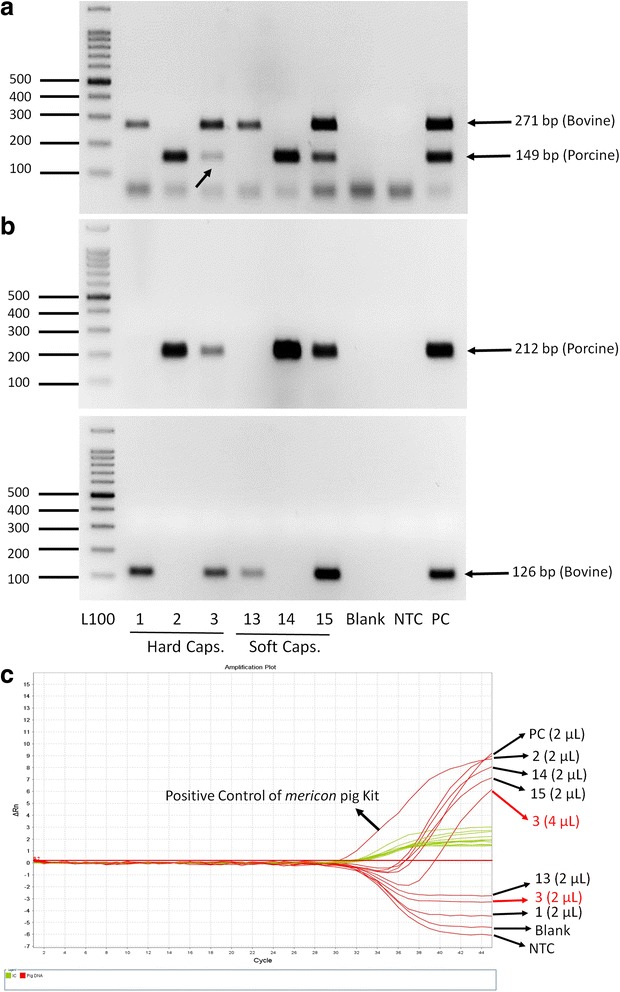
**a**: Agarose gel electrophoresis of duplex PCR products (149 bp for porcine and 271 bp for bovine mtDNA) resulting from DNA extraction (2 μL/20 μL PCR reaction) of three hard (no. 1, 2 and 3) and three soft (no. 13, 14 and 15) pharmaceutical gelatin capsules (The numbers are in accordance with the numbers in Table 3). L100: 100 bp ladder. Blank: Negative control of DNA extractions; NTC: Negative control of PCR reactions; PC: Positive control (Bovine/porcine powder mixture); the faint bands are marked with arrows. **b**: Agarose gel electrophoresis of simplex PCR products (212 bp for porcine and 126 bp for bovine mtDNA) of above DNA samples (2 μL) for confirming duplex PCR results. **c**: Amplification plot (Delta Rn *vs* Cycle) of real-time PCR of the above DNA samples (2 μL for number 1, 2, 13, 14 and 15–2 and 4 μL for number 3) using *mericon* pig kit for confirmation of Duplex PCR results. Red and green curves display target DNA (porcine) and internal control (IC), respectively

In the preliminary phase of this experiment, simplex PCR assays with porcine-specific (149 and 212) and bovine-specific (126 and 271) primers were used. As shown in Fig. 1a and Table 2, the simplex PCR detected as little as 0.1% porcine DNA (149 bp). The simplex PCR resulting in 212 bp porcine DNA products was used for confirmation and the same sensitivity was observed for detection of 212 bp porcine (0.1% porcine) DNA (Fig. 1b, Table 2). Also, as shown in Fig. 2b and Table 3, the gel electrophoresis of the simplex PCR amplified products showed expected bands of 212 bp for porcine and 126 bp for bovine gelatin capsules (confirmation of duplex PCR results in Fig. 2a).

### Duplex PCR

In this study, the duplex PCR was used to amplify two different bovine and porcine DNA sequences simultaneously (in one reaction mixture at the same time). The primers (four sets) were used pairwise (porcine/bovine: 149/271, 149/126, 212/271, 212/126) in various reaction conditions (i.e., different primer or Mg^2+^ concentrations, different annealing temperatures/times, different cycles) for selection and optimization of the most suitable primer pairs for acceptable duplex PCR amplification (data not shown). Only one pair (149-F/-R porcine and 271-F/-R bovine) showed acceptable results (i.e., sharp specific bands). After optimizing the technique using the reference binary porcine/bovine gelatin powder mixtures, it was possible to detect the presence of as little as 0.1% porcine DNA in the mixtures (Fig. 1a and Table 2) as well as in the gelatin capsules (Fig. 2a and Table 3), by using simultaneous amplification of mitochondrial cyt b for porcine and bovine DNA. Also, as shown in Fig. 1a it was possible to obtain acceptable normalized band intensity for porcine DNA [N_porcine_ = I_porcine_/(I_porcine_ + I_bovine_)] in bovine-porcine mixtures. By decreasing the number of PCR cycles from 40 to 37 and subsequently to 35, decreasing the concentration of 271-F/-R bovine primers from 0.25 to 0.15 and subsequently to 0.125 μM, and decreasing the concentration of 149-F/-R porcine primers from 0.4 to 0.35 μM, it was possible to ration (0.0, 0.08, 0.17, 0.28, 0.56, 0.78 and 1.0 for 0.0, 0.1, 1, 10, 50, 75 and 100% w/w of) porcine (DNA) contamination, respectively (with a sensitivity of 0.1%).

### Real-time PCR

In order to confirm the reliability and sensitivity of duplex PCR, all DNA samples from the standard gelatin powders and the capsules were evaluated using a commercial porcine DNA detection kit. As shown in Figs. 1c and 2c, amplification of internal control (green curves) was positive in all samples (no failed PCR) while the amplification of target porcine DNA (red curves) was negative in negative controls (blanks and NTC; no contamination in DNA extraction process and PCR reactions). The amplification of porcine DNA was positive in positive controls [porcine DNA provided in the kit and DNA extracted from 50% (w/w) bovine/porcine gelatin powder mixture]. As shown in Fig. 1c and Table 2, the real-time PCR amplification of porcine DNA in bovine-porcine gelatin powder mixtures (2 μL extracted DNA per 20 μL PCR reaction) was positive in presence of 10-75% (w/w) porcine DNA in the mixture, but was negative in 0.1 and 1% porcine (DNA) contamination. By increasing the extracted DNA volume to 4 μL (in 20 μL PCR reaction), the results became positive in samples containing 1% porcine DNA but the results did not become positive in samples with 0.1% porcine DNA even after increasing the volume to 9.6 μL (half the total volume of PCR reaction). The results of porcine DNA detection in 6 gelatin capsules (3 hard and 3 soft) by the commercial kit are shown in Fig. 2c and Table 3. The detection results of the other 18 gelatin capsules are shown in Table 3. The results for most samples are in agreement with those obtained from simplex- and duplex- PCRs. However, for two samples (no. 3 and 9) with faint porcine DNA bands detected on conventional PCR, the results were negative with 2 μL extracted DNA (per 20 μL PCR reaction) and became positive after increasing the concentration of the extracted DNA by two (no. 3) to five (no. 9) folds (4 and 9.6 μL).

## Discussion

Identifying the origins of animal products used in pharmaceuticals is a challenge for drug control laboratories and halal agencies. Particularly, Muslim countries seek to identify the presence of any forbidden (non-halal) ingredients in food products, pharmaceuticals, and beauty supplies [28–30]. Therefore, there is a need for reliable, quick, and highly sensitive methods to detect presence of such substances (e.g., swine products). In Muslim countries, gelatin used in food/pharmaceutical industries is mainly derived from bovine source. Thus, it is important to detect any possible contamination of bovine gelatin with porcine gelatin. Gelatin is a highly processed protein product, usually extracted from the skins, bones and connective tissues of animals (i.e., pig, cattle, fish, horses or poultry). As the result of methods used in gelatin production and processing, gelatin generally contains only very low amounts of highly degraded DNA (originated from animal cells). Since PCR-based techniques (using species- specific primers) are effective in identifying small pieces of DNA, they have received significant attention in recent years. The high copy number of mitochondrial DNA per cell and their probable stability under different processing conditions ensure amplification of expected PCR products even in samples containing small amounts of DNA [31]. However, it should be noted that the essential prerequisite for PCR amplification is to obtain sufficient amount of high-quality extracted DNA for analysis. In this study, after optimized DNA extraction, DNA extracts from 200 mg of minced gelatin capsule shells (from pharmaceutical capsules containing drug) had acceptable yield and quality to undergo further analysis by PCR. This suggests the adequacy of the used extraction protocol for gelatin capsules. Subsequently, we developed a duplex PCR assay (with 149 porcine-specific primers and 271 bovine-specific primers) for simultaneous detection of porcine and bovine DNAs. To overcome variations that might occur during DNA extraction, amplification and gel preparation, the intensity of the target band was normalized. By this developed/optimized duplex PCR, porcine gelatin content (as pure porcine gelatin samples or as impurity/contamination in bovine gelatin sample) as low as 0.1% was detected. Because of contradictory reports on the specificity of primers in species-specific detection studies, we used two additional methods to confirm duplex PCR. In order to verify the selectivity and sensitivity of this technique, we used simplex PCR with bovine- and porcine-specific primers; additionally, we used a commercially available porcine DNA detection kit (based on TaqMan quantitative real-time PCR). Shabani et al. used simplex PCRs with 212 porcine- and 271 bovine-specific primers (separately) for detection of porcine and bovine DNAs in gelatin, gelatin-containing foods, and capsule shells [8]. According to their results, as little as 0.1% w/w of porcine and bovine gelatin was detected using this technique. Our simplex PCR results from the same primers were consistent with their results. Soares et al. used duplex PCR for simultaneous detection of porcine (with 149 porcine-specific primers) and poultry (with 183 bp poultry-specific primers) DNA in meat [15]. They reported the detection of 149 bp porcine DNA with a sensitivity of 0.1%. Our simplex and duplex PCR results using the same primers for porcine DNA (149 bp) were consistent with their results. In 2005, Tasara et al. reported that conventional PCR assays targeting the subunit 8 of mitochondrial ATP synthase (ATPase8) in several gelatin samples successfully detected bovine DNA without any cross-reactivity with gelatin samples from other animals [26]. We used the same primers for verification of duplex PCR and our results were in agreement with theirs. Also, we used the commercial porcine DNA detection kit (based on quantitative real-time PCR) for verification of duplex PCR. Although the commercial kit confirmed the results of the conventional duplex and simplex PCR in most of our samples, its sensitivity was 1% for porcine DNA, which was lower than the 0.1% sensitivity of our semi-quantitative duplex PCR method. The commercial kit detected porcine DNA by increasing the amount of DNA extract by 2 or 5 folds. In 2012, Sahilah et al. compared two commercial PCR-based kits for detection of porcine DNA in pharmaceutical capsules and showed that the detection levels of those kits varied [4]. Our results clearly showed that conventional duplex PCR was sensitive enough for detection of considerably low percentages of bovine and porcine gelatin.

PCR-restriction fragment length polymorphism (RFLP) [21] and real-time PCR [6, 22] techniques are used for identification of gelatin capsules. Compared to PCR-RFLP (3-step process: PCR reaction, enzymatic digestion of PCR products and electrophoresis of digestion products), duplex PCR (2-step process: PCR reaction and subsequent electrophoresis of PCR products) can simultaneously detect the presence of porcine and bovine DNAs (amplification of both products in one reaction mixture at the same time) in very small samples (200 mg) more rapidly (needs fewer steps). Moreover, the latter is more cost effective (needs less reagents). Real-time PCR is sensitive and specific enough to trace small amounts of target DNA. However, due to the high cost of real-time equipment and reagents, not all laboratories are able to apply this method. Although the sensitivity of multiplex PCR amplification is known to be lower than that of simplex PCR amplification, using optimized duplex PCR we reached the same level of sensitivity (0.1%) for detecting porcine DNA as we had with simplex PCR. On the other hand, our results were comparable and even more sensitive than those obtained with the use of expensive real-time PCR-based commercially available (only porcine DNA detection not bovine) kit. It should be noted that PCR-based techniques (product size limit) are not suitable for the identification of very short DNA targets (15–30 bp), which can survive even under the harshest conditions of tissue processing [32]. According to our results, DNA fragments longer than 100 bp can be easily amplified using conventional simplex and duplex PCR techniques described in this study. Considering the limitations of this study, future studies are required to evaluate more diverse gelatin-based pharmaceutical products.

## Conclusion

In this study, the conventional duplex PCR methodology (semi-quantitative) proved to be a reliable and sensitive tool for detecting porcine DNA fragments (longer than 100 bp) present in hard- and soft-gelatin capsule shells (even in capsules containing drug) with a sensitivity of 0.1% using a 35-cycle duplex PCR. It means that using this technique, we can detect as little as 0.1% porcine DNA contamination/impurity in bovine gelatin capsules. The proposed methodology is an easy-to-follow, inexpensive, reliable, and sensitive alternative to expensive commercial detection kits, used for monitoring of food and pharmaceutical products.

## Abbreviations

ABI: Applied biosystem instruments
ATPase 8: The subunit 8 of mitochondrial ATP synthase
cytb: Cytochrome b
IC: Internal control
mtDNA: Mitochondrial DNA
NTC: No template (DNA) control
PC: Positive control
PCR: Polymerase chain reaction
RFLP: Restriction fragment length polymorphism
TAE: Tris–acetate–ethylene diamine tetra acetic acid
UV: Ultraviolet
